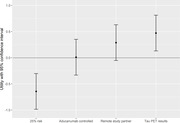# Utility of recruitment incentives in early Alzheimer’s disease clinical trials

**DOI:** 10.1002/alz.092808

**Published:** 2025-01-09

**Authors:** Marina Ritchie, Megan G. Witbracht, Eunji Russ, S. Ahmad Sajjadi, Gaby Thai, Steven Tam, Daniel L Gillen, Joshua D Grill

**Affiliations:** ^1^ University of California, Irvine, Irvine, CA USA

## Abstract

**Background:**

Amid recent approvals, early Alzheimer’s disease (AD) remains an active area of treatment development, but research on the utility of recruitment incentives in early AD trials remains limited. We examined how trial design features impact enrollment decisions among Mild Cognitive Impairment (MCI) patients and their family members.

**Method:**

We performed a conjoint analysis experiment to compare early AD patients’ preferences for trial features. Individuals with MCI were asked to rate their willingness to participate in a series of trial scenarios (using a 7‐point ordered scale) that varied in four pre‐selected factors, each with two levels. Three trial incentives were included: 1) active‐control design with participants randomized to an investigational anti‐amyloid therapy or aducanumab (vs. placebo control), 2) tau PET disclosure (vs. no return of tau PET results), and 3) remote study partner participation (vs. required in‐person study partner participation). We included one negative control (5% vs. 25% risk of brain swelling). We used a generalized estimating equations (GEE) model to account for within‐subject correlation and to assess the utility of the differing factor levels. An exchangeable working correlation structure was used for parameter estimation, and robust variance estimates for model coefficient estimates were used for final inference.

**Result:**

Among 26 patients with MCI (mean [SD] age 78.3 [8.2], 31% female, 85% non‐Hispanic White), returning tau PET results had the highest utility (est: 0.47; CI: 0.13, 0.81; p = 0.007). Trials with the option for remote study partner participation showed a similar trend in utility (est: 0.29; CI: ‐0.05, 0.63; p = 0.097). Trials with active‐controlled design (est: 0.01; CI: ‐0.33, 0.35; p = 0.956 did not demonstrate significant utility and trials with higher risk of brain swelling had a negative utility (est: ‐0.64; CI: ‐0.99, ‐0.30; p<0.001) (Figure 1).

**Conclusion:**

These findings suggest that returning biomarker results may have a greater impact on participant enrollment decisions than would use of active‐controlled designs or providing the option for study partners to participate remotely.